# Effects of Interoceptive and Exteroceptive Attention Training on Desire-Driven Eating in Healthy Adults: A Quasi-Experimental Early-Stage Feasibility Study

**DOI:** 10.3390/foods14234078

**Published:** 2025-11-27

**Authors:** Chanette Frederiksen, Derek Victor Byrne, Barbara Vad Andersen

**Affiliations:** 1 Food Quality Perception and Society Team, iSense Lab, Department of Food Science, Faculty of Technical Sciences, Aarhus University, 8000 Aarhus, Denmark; derekv.byrne@food.au.dk (D.V.B.); barbarav.andersen@food.au.dk (B.V.A.); 2 Sino-Danish College (SDC), University of Chinese Academy of Sciences, Beijing 101408, China

**Keywords:** interoception, exteroception, snacking behaviour, eating behaviour, cravings, desires, unhealthy snacks, snack foods

## Abstract

Interoceptive and exteroceptive awareness (awareness of bodily signals and sensory experiences) are increasingly recognised as important for guiding eating behaviour. However, research remains limited on how such awareness can be enhanced in everyday contexts and how this affects eating behaviour. This study explored whether an interoceptive-exteroceptive attention training programme, delivered through written instructions, could enhance such abilities in healthy adults and promote healthier food choices. Thirty-five healthy adults completed a 14-day attention training period involving daily use of written materials designed to enhance attention to interoceptive and exteroceptive cues before, during, and after eating. Measures included objective and self-reported indicators of interoception, exteroception, and dietary behaviour, assessed pre- and post-attention training. Participants reported significant increases in attention to sensory experiences and intuitive and mindful eating. A reduction in the consumption of unhealthy snack components was also observed. In post-study evaluations, participants described the attention training as positive and awareness-enhancing. Most reported becoming more attentive in their eating and snacking behaviour, and over 80% intended to continue practising awareness after the study. While not all outcomes reached statistical significance, the findings provide preliminary, proof-of-concept evidence supporting the feasibility of interoceptive-exteroceptive attention strategies for fostering healthier, more self-regulated eating in everyday life.

## 1. Introduction

Today, in many parts of the world, food is abundant, and eating has increasingly become a source to pleasure, comfort, and satisfaction of desires [1]. As a result, eating behaviour is increasingly guided by hedonic aspects (e.g., pleasure, wanting, and liking) beyond homeostasis (i.e., obtaining energy for survival and maintaining bodily functions) [2,3]. Experiencing pleasure from eating, whether derived from social company, the physical environment, or the food itself, is not inherently problematic [2,4]. However, eating behaviour can be of concern when it is excessively driven by pleasure-seeking behaviour. This includes instances where individuals experience strong cravings for specific foods, that is, a strong desire, urge, or wanting to eat [5]. Food cravings are widely recognised as a challenge for individuals attempting to modify their eating patterns [6,7] and have been shown to prospectively predict increased energy intake and subsequent weight gain over time [8]. In contrast, previous research shows that more internally guided eating patterns, including intuitive eating, are associated with beneficial health outcomes such as lower BMI, reduced disordered eating and improved psychological well-being [9,10,11,12]. At the same time, everyday eating often occurs under conditions of distraction and multitasking (i.e., watching television, driving or working), which can impair satiety perception and promote food intake [13,14,15,16,17]. In contrast, studies indicate that more attentive forms of eating, including focused engagement with sensory properties of food, are linked to healthier dietary patterns, including reduced snack consumption [18,19,20,21]. Alongside these behavioural challenges, eating patterns have shifted considerably. Snacking now accounts for nearly one-third of daily energy intake, and a significant proportion of these snacks are energy-dense and low in essential nutrients (e.g., candy-like snacks) [22]. Their frequent consumption has been linked to an increased risk of overweight and obesity due to their contribution to a sustained positive energy balance [23,24].

Various interventions and strategies (e.g., public health campaigns, official dietary guidelines, structured dietary programmes) have been developed in an attempt to support healthier and more sustainable eating behaviour [25]. However, many of the available strategies rely on restriction-based approaches, with limitations for when, how much, and what to eat, and often yield only short-term results, probably because they do not address the underlying factors influencing the individual’s eating behaviour [26,27]. On top of this, many people struggle to maintain behavioural change strategies over time [6], highlighting the need for more research into approaches that support long-lasting change. A growing body of research advocates for alternative, holistic, and health-oriented approaches to dietary behaviour change, that expand the focus beyond restriction and weight loss as the primary means of improving health [26]. Interoceptive and exteroceptive strategies may represent an approach to supporting healthier eating behaviours, not by imposing restrictions but by fostering greater awareness [28,29]. Interoception involves attending to internal bodily states such as hunger, fullness, and pleasure [2,30], while exteroception refers to the perception and processing of sensory information originating from outside the body, typically through receptors located in the skin, eyes, ears, nose, and mouth [31]. In the context of eating behaviour, exteroceptive food-related sensory cues include visual, olfactory, gustatory, auditory, and somatosensory inputs. To avoid conceptual confusion, exteroception is not used here in the sense of external eating or food cue reactivity, which typically describes automatic, cue driven responsivity to environmental stimuli. Though distinct, interoceptive and exteroceptive processes interact and together provide a foundation for regulating eating behaviour [19,30,32]. Importantly, framing eating behaviour through interoceptive and exteroceptive processes may help to specify underlying perceptual and regulatory mechanisms [28,33]. Mindful and intuitive eating, by contrast, represent broader approaches that implicitly build on interoceptive and exteroceptive functioning [9]. Both approaches have been linked to beneficial outcomes, including reduced disordered eating and improved well-being, yet findings across dietary and health outcomes remain inconsistent [33,34]. As Tapper (2022) [33] highlights, a wide range of processes could underline the influence of mindfulness-based practices on eating, but much of the current evidence remains speculative. One possible reason for this inconsistency is that the underlying mechanisms are rarely specified in detail, further complicated by the fact that many of these interventions combine multiple components, making it difficult to determine what actually drives the observed effects [33]. Without a more precise understanding of these mechanisms, it is difficult to identify which elements are most effective, how they should be adapted to different eating-related challenges, or whether some practices might even prove counterproductive in certain contexts (e.g., weight loss interventions).

By explicitly focusing on perceptual and self-regulatory processes of eating—such as interoceptive and exteroceptive attention—it may be possible to design strategies that strengthen individuals’ ability to sense, interpret, and act on bodily and sensory cues [28]. This emerging mechanistic perspective helps to clarify the conceptual overlap with mindful and intuitive eating, while also offering a potential pathway toward more precise, testable, and scalable approaches to supporting healthier eating behaviour. Previous research by Palazzo and colleagues (2022) has demonstrated that interoceptive and exteroceptive abilities can be enhanced [29]. In their research, participants engaged in four 2 h workshops over a period of 3 to 4 weeks, supplemented by inter-session home exercises. From a feasibility perspective, however, it is also important to examine early-stage methods that require less structured time commitment and can be seamlessly integrated into everyday routines, thereby offering a potentially more accessible approach for a wider range of individuals. In a previous study, it was demonstrated that it is possible to direct attention towards interoceptive-, exteroceptive-, and a combination of interoceptive and exteroceptive signals while eating by providing written instructions [35]. Although the study did not detect an effect on momentary rating on appetite, pleasure and sensory-specific desires, it was hypothesised that this may be due to the study’s design, which included a single-session intervention and thus limited exposure to the attention strategy. Since dietary habits develop over longer timeframes [36,37], it is important to explore whether prolonged interoceptive-exteroceptive attention training can yield more sustained changes in eating behaviour.

The overall aim of this study was to investigate whether a 14-day interoceptive–exteroceptive attention training programme, delivered through written instructions, could enhance interoceptive and exteroceptive abilities in healthy adults and promote healthier food choices. Specifically, the objectives were to study how the interoceptive-exteroceptive attention training affected: (1) interoceptive and exteroceptive abilities, (2) desire-driven eating, particularly regarding snack food consumption, and (3) the experience of the attention training period.

In the present study, attention training is defined as the systematic, repeated practice of directing attention toward interoceptive (bodily) and exteroceptive (sensory) cues before, during, and after eating episodes. It was hypothesised that a 14-day interoceptive–exteroceptive attention training programme, delivered through flexible and easy-to-use written materials, would enhance attentional focus on bodily (interoceptive) and sensory (exteroceptive) cues and reduce desire-driven eating, particularly the consumption of unhealthy snacks. Finally, it was hypothesised that the study would demonstrate potential for real-world implementation via participants’ experiences with the attention training materials. The hypotheses build on evidence from previous studies, including systematic reviews, indicating that increased attention to internal and external eating-related cues might support more internally regulated eating patterns and healthier food choices [32,33]. By supporting participants in becoming more attuned to their bodily sensations and sensory experiences during eating, the attention training was expected to promote greater self-regulation and reduce unhealthy snack consumption.

## 2. Materials and Methods

### 2.1. Study Design

**Figure 1:**
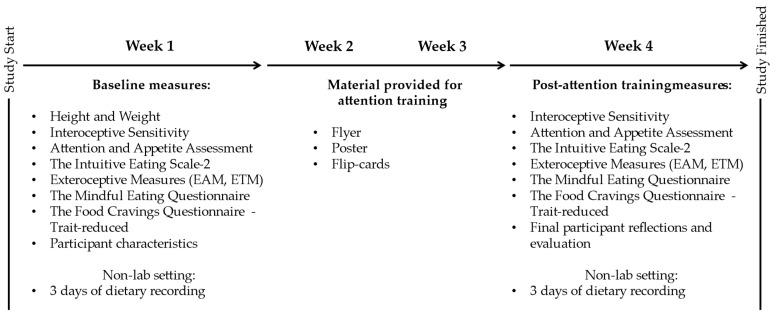
Study timeline and measurement.

The study was conducted as an early-stage feasibility, quasi-experimental study employing a single-group pre–post design to explore preliminary signals of change and assess procedural feasibility. The study took place across approximately four weeks. Week 1 included baseline measurements. Weeks 2 and 3 consisted of a 14-day structured attention training period carried out in participants’ everyday environments. Week four included the post-attention training measurements in which participants repeated the measurement series. Outcomes were evaluated by comparing data from week 1 (baseline data) with data from week 4 (post-attention training data), with participants serving as their own controls. A detailed description of the study procedure can be seen in Section 2.3, and a visual overview is provided in Figure 1.

### 2.2. Participants, Recruitment and Pre-Screening

To ensure sufficient statistical power for the present study, a power analysis was conducted using R Studio, version 4.4.1 (Boston, MA, USA), based on a previous study with a comparable design [29]. This study examined the effects of interoceptive versus exteroceptive attention on interoceptive sensitivity and exteroceptive expression. The analysis was based on an expected effect size (delta) of 0.08, a standard deviation (sd) of 0.155, a significance level (α) of 0.05, and a desired statistical power of 0.80. Given the within subject design, a paired t test was specified and a two-sided hypothesis was applied. The calculation indicated that a sample size of 32 participants would be required to detect an effect of the expected magnitude with 80% power, assuming a 5% risk of Type I error.

To allow for a potential minor dropout, a total of 40 healthy Danish citizens were recruited. Recruitment was done through a participant subject pool at Aarhus University, as well as via social media platforms (e.g., Facebook and LinkedIn) and a national research recruitment website (www.trialtree.dk). A pre-screening process ensured that participants met the inclusion criteria of: being aged between 20 and 59 years, not being pregnant or breastfeeding (self-reported), and having a BMI of at least 18.5 (with no upper limit). Additionally, participants could not suffer from metabolic (e.g., diabetes), endocrine (e.g., osteoporosis), psychiatric (e.g., depression), or neurological (e.g., dementia) disorders or diseases, nor from any eating disorders, be on a very restrictive diet, or have any form of substance abuse or drug addiction. Smokers and users of nicotine products were also excluded, as were individuals with a nutritional profession involving dietary guidance (e.g., dietitians) and individuals with small children living at home. Participants were required to read, write, and speak Danish, as the study was conducted in the Danish language. Five out of the 40 recruited participants dropped out during the study due to sickness, busy schedules or lack of motivation, leaving 35 participants who fully completed the study. Participant characteristics are presented in Table 1. Participant data were anonymised prior to analysis through the removal of all personal identifiers and replacement with numerical codes. Only aggregated results are reported.

### 2.3. Study Procedure

The study was conducted between November 2024 and March 2025 and took place across approximately four weeks.

Week 1 and 4: Baseline and post-attention training measures: The baseline measurements in week one and the post-attention training measurements in week four were conducted at the sensory lab facilities at the Department of Food Science, Aarhus University, Denmark. Participants were instructed to arrive well rested and to have fasted for at least two hours prior to the visit. Adherence was confirmed verbally upon arrival, although food intake prior to arrival was not otherwise recorded. While no further control of dietary intake was implemented, potential differences in hunger, satiety or metabolic state across test days were accounted for in the statistical analyses (see Section 2.5.1).

Uniquely in week one, participants provided written informed consent before taking part in the study. Afterwards, height and weight were measured using professional, medically approved equipment from TANITA [38], and participants completed a short set of questions regarding participant characteristics (see Section 2.4.4).

In both weeks, interoceptive sensitivity was measured (see Section 2.4.2) following a task designed to assess attention and appetite in a realistic eating situation (see Section 2.4.1). Finally, participants completed a set of established previously validated questionnaires together with additional self-developed items targeting interoceptive and exteroceptive eating behaviours (see Section 2.4.3).

In addition to the lab-based measurements, participants completed a three-day dietary registration on two weekdays and one weekend day, both immediately before and immediately after the attention training period in their own environment (see Section 2.4.5). This provided complementary data on potential changes in dietary intake across the study. Before leaving the sensory lab facilities in week one, participants received instructions and all necessary materials for the 14-day attention training, including guidance on how to complete the dietary registration.

Uniquely in week four, additional questions were asked regarding participants’ experiences with the study and the attention training period (see Section 2.4.4).

Week 2–3: Attention training period: In contrast to the measurements collected at baseline (week 1) and post-attention training (week 4), the attention training period took place in natural settings (i.e., non-laboratory environments), including participants’ homes and other everyday eating environments. The attention training period lasted 14 days, during which participants trained their attention to interoceptive and exteroceptive cues using written materials. The use of each material was standardised through explicit instructions provided to all participants. Inspired by a previous study [35], three written materials were developed for use during the 14-day attention training period. (1) A physical flyer (Supplementary Materials, Figure S1) to enhance participants’ knowledge of how to train interoceptive and exteroceptive abilities, including an explanation of these concepts. Participants were required to read the flyer on the first day of the attention training period and again after seven days. (2) A poster in physical as well as digital format (Supplementary Materials, Figure S2) to enable participants to quickly and easily be reminded of key aspects of interoception and exteroception. The poster included five key eating behaviour advice: Set the stage for a good eating experience, Consider the purpose of the meal, Sense the food, Sense your physical body, and Notice your mental sensations. For each of these five key aspects, a short explanatory text was included to engage participants. The participants were instructed to review the poster at least once per day. (3) Flip cards (Supplementary Materials, Figure S3, Table S1) presented the five key pieces of eating behaviour advice from the poster. Each advice item was accompanied by additional elaboration cards providing more specific guidance. For example, for the guideline on “Sense the food”, the elaboration encouraged participants to notice how the food looked, smelled, felt in the mouth, tasted, and sounded when chewed, as well as to eat slowly to perceive these characteristics. The participants were asked to use these flip cards during one main meal every second day. Attention training was thus structured as a daily activity, supported by standardised instructions on how and when to use each material, to promote consistency. The content of the materials specifically addressed bodily sensations (e.g., emotions) and sensory qualities (e.g., sweet, salty, or fatty tastes) often linked to unhealthy snacking [39,40], aiming to increase participants’ awareness of their internal and external responses in tempting eating situations and to support more deliberate, need-based snack choices. This duration of 14 days was selected to provide repeated exposure to the attentional instructions, based on theoretical frameworks suggesting that sustained and repeated attentional engagement may be necessary to facilitate awareness and behavioural change [41], while also considering the timeframe to be manageable for the participants. Previous studies have used similar timeframes to examine behavioural and perceptual changes following attentional or mindful eating interventions [42,43].

### 2.4. Measurements

In order to test the effect of the interoceptive exteroceptive attention training, measurements in week one (baseline) and week four (post attention training) were collected. A detailed description of the measures included is presented below.

#### 2.4.1. Attention and Appetite Assessments in a Realistic Eating Situation

To assess participants’ attention to interoceptive and exteroceptive cues and subjective appetite-related sensations, participants rated appetite- and sensory-specific desires before and after eating a test meal. Gathering data during an actual eating situation allowed for a more contextually grounded and experience-near understanding of participants’ cognitive and sensory engagement with food, compared to hypothetical or retrospective assessments. Importantly, participants did not receive any instructions on what to do or pay attention to while eating. The test meal consisted of a portion of skyr with toppings and 150 mL of water. The ratings included hunger, fullness, and desire for something ‘sweet’, ‘salty’, ‘fatty’, ‘spicy’, ‘sour’, or ‘bitter’ and were recorded using a 100 mm computerised Visual Analog Scale (VAS), anchored from 0 = ‘Not at all’ to 100 = ‘Extremely’. The items were inspired by the Five Factor Satiety Questionnaire [44]. See Supplementary Materials, Table S2, for the full item list.

After completing the post-meal ratings, participants were asked to describe freely, in their own words, the focus of their attention during the test meal. This open-ended expression task, adapted from previous dietary interventions [29,45], is based on the assumption that expression of experience presupposes conscious awareness [46]. Moreover, the number and nature of terms used have been suggested to reflect cognitive engagement and emotional involvement [47]. The task was used to assess the focus of participants’ attention during the test meal and was included to evaluate whether participants’ ability to focus attention on the eating experience (exteroceptive and interoceptive cues) and register appetite-related sensations and sensory-specific desires changed following the attention training period. The measurement was conducted at baseline and post-attention training to facilitate the calculation of differences.

#### 2.4.2. Interoceptive Sensitivity

To objectively assess interoceptive sensitivity, a Heartbeat Tracking Task (HTT) was used. The task followed the original methodology developed by Schandry (1981) [48] and applied by Palazzo and colleagues (2022) [29]. In brief, the procedure involves participants counting their heartbeats over four intervals of 25, 35, 45, and 55 s, with a 30 s rest interval in between. Their counted beats are compared with values recorded by an oximeter. A cardiac perception score is calculated as the mean accuracy across the four intervals, with scores closer to 1 indicating greater interoceptive sensitivity:

$$Score=\;1-\frac{\left(recorded\;heartbeats-counted\;heartbeats\right)}{recorded\;heartbeats}$$

The measurement was conducted at baseline and post-attention training to facilitate the calculation of differences.

#### 2.4.3. Questions and Questionnaires

To examine the effect of attention training on interoceptive and exteroceptive abilities, as well as desire-driven eating, participants completed a series of questionnaires: The Intuitive Eating Scale, Exteroceptive measures, the Mindful Eating Questionnaire, and the Food Cravings Questionnaire, which are explained in the following sections. All questionnaires were answered at baseline and post-attention training.

The Intuitive Eating Scale: The Intuitive Eating Scale-2 (IES-2) [49] was used to assess subjective self-reports of intuitive eating [50]. The scale captures key aspects of interoceptive eating, including reliance on internal hunger and satiety cues, eating for physical rather than emotional reasons, and unconditional permission to eat. Responses were recorded on a 5-point Likert scale (1 = ‘Strongly disagree’ to 5 = ‘Strongly agree’). Final mean scores ranged from 1 to 5, with higher scores indicating a greater tendency toward intuitive eating.

Exteroceptive Measures: As no validated questionnaire currently exists to assess self-reported attention to and trust in sensory cues, a complementary set of exteroceptive items was developed in a previous study [35] to parallel the interoceptive focus of interoceptive measures. Specifically, the items were designed to capture how participants attend and interpret external sensory food cues during eating. Two types of measures were included: Attention to sensory stimuli, referred to as Exteroceptive Attention Measures (EAM), which were adapted to focus on external sensory input. Items covered attention to taste, texture, smell, appearance, temperature, sounds, combined sensory properties, and the ability to remain focused on these cues without distraction. Responses were recorded using a 100 cm computerised VAS, anchored from 0 = ‘Not at all attentive’ to 100 = ‘Extremely attentive’, with higher scores indicating greater sensory attention. Trust in sensory desires, referred to as Exteroceptive Trust Measures (ETM), was inspired by trust and reliance dimensions of the IES-2. These items measured participants’ confidence that their sensory food desires reflect actual bodily needs. Responses were recorded on a 5-point Likert scale (1 = ‘Strongly disagree’ to 5 = ‘Strongly agree’), with higher scores indicating greater trust in the statement. While previous research has linked exteroceptive attention to improved eating attitudes and behaviours [29,45], a standardised instrument for measuring these constructs is lacking. The developed items are presented in Supplementary Materials, Table S2.

The Mindful Eating Questionnaire: The Mindful Eating Questionnaire (MEQ) [51] was used to assess broader changes in participants’ self-reported eating behaviour related to present-moment awareness and emotional regulation during eating. The MEQ is a psychometric tool designed to measure five dimensions of mindful eating: disinhibition (tendency to eat in response to external or emotional cues), awareness (conscious attention to eating), external cues (susceptibility to food-related environmental stimuli), emotional response (emotional eating tendencies), and distraction (eating while engaged in other activities). This questionnaire evaluates individual differences in the tendency to eat mindfully across various situations. Responses were recorded on a 4-point Likert scale (1 = ‘Never/Rarely’ to 4 = ‘Usually/Always’), with an additional response option for some questions (0 = ‘Not applicable’). Final mean scores ranged from 1 to 4, with higher scores indicating a greater tendency toward mindful eating.

The Food Craving Questionnaire: The Food Cravings Questionnaire–Trait–Reduced (FCQ–T–r) [52], was used to assess participants’ self-reported craving traits. The FCQ-T is a psychometric tool assessing stable, trait-like aspects of food cravings, capturing individual differences in their frequency and intensity across situations and over time [53]. Responses were recorded on a 6-point Likert scale (1 = ‘Never/not applicable’ to 6 = ‘Always’). The final scores from the three questions ranged from 15 to 90, with higher scores indicating a higher level of craving traits. A score of 50 or above may indicate clinically relevant levels of food cravings [52].

#### 2.4.4. Questions Administered Only Once

Besides the questionnaires mentioned above, questions regarding participant characteristics were asked at baseline (week 1), including gender (single-forced-choice), age (in years), physical activity habits (single-forced-choice), highest completed educational level (single-forced-choice), and employment status (single-forced-choice); see Supplementary Materials, Table S2. In week 4 (In post-attention training measurements), additional questions about participants’ reflection and evaluation of the attention training period were asked: (1) overall experience of participating in the study, (2) general perceived changes in overall eating behaviour, (3) general perceived changes in snacking behaviour, and (4) the likelihood of continuing to practice awareness and attention beyond the study. Questions were answered via open-ended reply fields, and participants were encouraged to elaborate on their answers. The aim was to assess perceived changes in eating behaviour, particularly in relation to snacking, and to evaluate the potential for long-term implementation of interoceptive-exteroceptive strategies in everyday life. The full set of questions is provided in Supplementary Materials, Table S2.

Finally, to assess adherence and perceived supportiveness of the attention training materials, participants indicated how often they had used each material (single forced choice) and how supportive they found each material during the first and last week of the attention training period. For example: “In the first week, how helpful/supportive did you find the flip-cards?” Each item was presented in four versions (two per material, assessing week 1 and week 2 separately) and rated on a 100 cm computerised VAS (0 = not supportive at all, 100 = extremely supportive). Results are provided in Supplementary Materials, Table S3. No statistical analyses were conducted, as these questions served only as a procedural check.

#### 2.4.5. Dietary Records

To examine whether the attention training period affected desire-driven eating, particularly in relation to snack food consumption, including unhealthy snacks, participants completed a three-day dietary recording (comprising two weekdays and one weekend day) at both baseline and after the attention training period. Participants were instructed to record everything they consumed, excluding water, by providing detailed written descriptions of all foods and drinks. The study staff provided a recording template to support consistent documentation.

### 2.5. Data Analysis

All variables underwent initial descriptive analysis using Microsoft Excel^®^ (Microsoft, 2503, Redmond, WA, USA). Subsequent data analyses were conducted using XLSTAT^®^ (version 2024.2.2, Addison, New York, NY, USA), with a significance level set at α < 0.05 for all statistical calculations.

#### 2.5.1. Attention and Appetite Assessment in a Realistic Eating Situation

To examine whether the attention training period influenced participants’ in-the-moment awareness of appetite-related sensations and focus on interoceptive and exteroceptive cues, both quantitative and qualitative analyses were conducted based on data collected during the test meal at baseline and post-attention training.

Changes in subjective appetite and sensory-specific desires were analysed using delta values (Δ) reflecting the difference between pre-meal and post-meal ratings. Specifically, Δ1 was calculated at baseline and Δ2 at post-attention training, with Δ representing the change from pre- to post-test meal consumption for each variable. Paired t-tests were used to assess whether there were significant differences between Δ1 and Δ2, indicating changes in appetite sensations or desire for specific taste qualities following the attention training period. Internal consistency of the appetite measures was assessed separately for pre-meal and post-meal ratings at both baseline and post-training using Cronbach’s alpha.

To analyse participants’ attentional focus during the test meal, written responses from the open-ended expression task were examined using qualitative content analysis [54]. Each response was segmented into meaning units, which were then coded and grouped into two overall categories: ‘interoceptive observations’ and ‘exteroceptive observations’. Interoceptive observations reflected awareness of bodily sensations (e.g., hunger, fullness, pleasure, enjoyment) or general physiological changes (e.g., temperature, movements). Exteroceptive observations included references to the sensory characteristics of the food (e.g., smell, appearance, taste, texture), its ingredients, or the act of eating itself (e.g., pace, rhythm). To ensure accuracy and consistency, two researchers independently reviewed all coded responses before finalising the analysis (blinded to time point: pre- or post-attention training). The frequency of meaning units in each category was calculated for each participant and compared between the two test days using paired t-tests to assess potential changes in attentional focus.

#### 2.5.2. Interoceptive Sensitivity and Questionnaires

To test for the effects of the attention training period, paired t-tests were conducted on the following measures: interoceptive sensitivity scores from the HHT, IES-2, EAM, ETM, MEQ, and FCQ–T–r. Internal consistency of the questionnaire measures was assessed at baseline and post-training using Cronbach’s alpha.

#### 2.5.3. Participant Reflections and Evaluation

Participants’ replies to questions about the experience of the attention training period were analysed using qualitative content analysis. Replies were segmented into meaning units and categorised, and chi-square tests were applied to examine the distribution of responses across categories. For the question about the overall experience of participating in the study, responses were divided into one or more of three main categories: (1) positive experience (e.g., “fun”, “interesting”, “inspiring”), (2) awareness-enhancing (e.g., “thought-provoking”, “attention-enhancing”, “useful”) and (3) challenging (e.g., “boring”, “hard to change habits”, “difficult to find time”). As a single response could reflect multiple experiences, it could be placed into more than one category. For the question about potential perceived eating behavioural changes and changes in snacking behaviour, respectively, responses were grouped into: (1) Yes—more attentive/better handling, (2) Yes—a little/maybe and (3) No—no change in attention or handling. For the question about the intention to continue practising attention training, responses were categorised as: (1) Highly probable, (2) Unsure/maybe, (3) Not likely. Each participant’s responses were coded accordingly, and the frequency of responses in each category was analysed using chi-square tests. An independent researcher reviewed all coding to ensure reliability.

#### 2.5.4. Dietary Records

To examine whether the attention training period influenced the healthiness of snack meals consumed between main meals, all recorded snack meals from before and after the attention training were reviewed, categorised, and counted. Each individual item within a snack meal was coded as either (1) healthy, (2) unhealthy, or (3) neutral, based on definitions provided by the Danish National Food Institute [55,56]. For example, if a participant reported eating both a piece of fruit and a chocolate bar in the same snack meal, the fruit was classified as healthy, and the chocolate bar was classified as unhealthy, regardless of the quantity. This means that a single snack meal could contain items from more than one category. To ensure accuracy and consistency in the classification, two researchers independently reviewed all items before finalising the coding (blinded to time point: pre- or post-attention training). For each participant, the total number of items in each category was calculated and analysed using a paired t-test.

## 3. Results

### 3.1. Participant Characteristics

A total of 35 healthy adults participated in the study, the majority of whom were female (71.4%). The participants had a mean age of 30 years (SD 11.45) and an average body mass index (BMI) of 24.82 (SD 4.62). Most were students (60%) and reported engaging in physical activity three to six times per week (62.9%). The sample had a varied educational background, with nearly half having completed a medium-length higher education programme (45.7%) (Table 1).

### 3.2. Attention and Appetite Assessment in a Realistic Eating Situation

To explore potential changes in attentional focus during eating, written responses from the open-ended expression task, completed after eating a test meal, were analysed and categorised into ‘interoceptive’ and ‘exteroceptive’ meaning units (Table 2). Results indicated that participants primarily directed their attention toward exteroceptive cues (e.g., taste, texture, appearance) both before and after the attention training period. At baseline, participants produced, on average, 104 exteroceptive meaning units, accounting for 81.3% of all coded observations. This number increased slightly to 123, meaning units (73.7%) after the attention training period, though this change was not statistically significant. Interoceptive meaning units (e.g., hunger, fullness, bodily sensations) increased from 24 (18.8%) at baseline to 44 (26.3%) post-attention training, suggesting a shift toward greater internal bodily awareness, although not reaching a significant difference.

**Figure 2:**
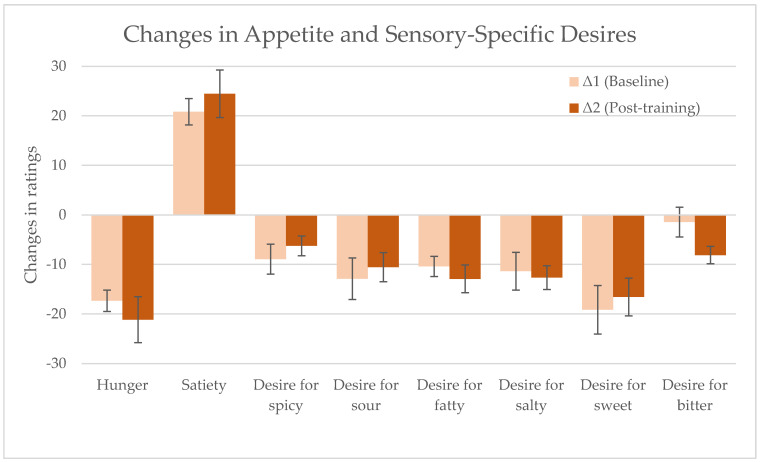
Change in hunger, satiety, and sensory-specific desires from pre- to post-test meal consumption at Baseline (Δ1) and pre- to post-snack post-attention training (Δ2). Note: Values are presented as means ± SEM.

To examine whether the attention training period influenced participants’ in-the-moment awareness of appetite and desire-related sensations, delta values from the test meal were calculated at baseline and post-attention training (Figure 2). Thus, Δ1 represents the change from pre- to post-test meal consumption at baseline (Δ1), whereas Δ2 represents the change from pre- to post-test meal consumption post-attention training (Δ2). No statistically significant differences were observed between Δ1 and Δ2 for any of the ratings. For means and standard deviation (SD) values for all ratings, see the Supplementary Materials (Table S4). Internal consistency of the appetite scales was also examined at each time point. At baseline, Cronbach’s α was 0.62 (standardised α = 0.65) before eating and 0.72 (standardised α = 0.76) after eating. A similar pattern was observed post-training, with α = 0.63 (standardised α = 0.72) before eating and α = 0.70 (standardised α = 0.78) after eating.

### 3.3. Interoceptive Sensitivity

Interoceptive sensitivity was assessed at baseline and post-attention training using the HTT [48], an objective behavioural measure, to assess whether the attention training period improved participants’ ability to perceive internal bodily signals. Results revealed no statistically significant change from baseline to post-training (Table 3).

### 3.4. Effect on Interoceptive and Exteroceptive Eating

To assess subjective changes in interoceptive eating behaviour, participants completed the IES-2 [49], at both baseline and post-attention training. Results revealed a statistically significant increase in overall intuitive eating scores from baseline (M = 3.20, SD = 0.63) to post-attention training (M = 3.49, SD = 0.51), *p* = 0.001, d = 0.62 (Table 4).

More detailed analyses of the IES-2 subscales showed significant improvements in both Eating for Physical Rather Than Emotional Reasons (EPR) and Reliance on Hunger and Satiety Cues (RHSC). EPR increased from baseline (M = 3.14, SD = 0.77) to post-attention training (M = 3.51, SD = 0.66), *p* < 0.001, d = 0.68. Similarly, RHSC increased from baseline (M = 3.11, SD = 0.87) to post-attention training (M = 3.57, SD = 0.92), *p* < 0.001, d = 0.65. In contrast, the Unconditional Permission to Eat (UPE) subscale showed a non-significant change from baseline (M = 3.57, SD = 0.70) to post-attention training (M = 3.77, SD = 0.73), *p* = 0.070, d = 0.32. This pattern suggests a tendency toward intuitive eating, although the change did not reach statistical significance. Internal consistency of the IES-2 was good in this sample, with Cronbach’s α of 0.84 (standardised α = 0.83) at baseline and 0.82 (standardised α = 0.82) post-attention training.

To explore whether the attention training period influenced participants’ exteroceptive attention during eating, changes in participants’ self-reported attention to and trust in sensory food characteristics were assessed using items from the EAM and ETM scales [35] (Table 5). Following the attention training period, participants significantly increased in several aspects of sensory attention. Specifically, attention to taste (*p* = 0.002, d = 0.65), texture (*p* = 0.040, d = 0.43), temperature (*p* = 0.042, d = 0.44), and chewing sounds (*p* = 0.005, d = 0.61) all increased significantly. In addition, attention to the combined sensory experience, reflecting attention of the interplay between taste, texture, smell, and appearance, also showed a significant increase (*p* = 0.022, d = 0.53). No significant changes were observed in attention to smell, appearance, or the ability to maintain undistracted attention to sensory properties. A non-significant change was observed for trust in sensory desires being guided by bodily needs (ETM), with scores increasing from baseline to post-attention training (*p* = 0.063, d = 0.29). Cronbach’s α indicated good to excellent internal consistency. For the EAM, α was 0.87 (standardised α = 0.87) at baseline and 0.86 (standardised α = 0.86) post-training. For the ETM, α was 0.83 (standardised α = 0.84) at baseline and 0.82 (standardised α = 0.82) post-training.

To assess broader changes in participants’ eating behaviour related to awareness and emotional regulation during eating, subjective ratings from the MEQ [51] were collected at both baseline and post-attention training. The MEQ provides a general measure of mindful eating tendencies, capturing participants’ self-reported ability to eat with awareness, manage emotional responses to food, and remain attentive and undistracted during meals. Overall MEQ scores increased significantly from baseline (M = 2.49, SD = 0.30) to post-attention training (M = 2.63, SD = 0.30), *p* = 0.008, d = 0.49, suggesting an overall improvement in mindful eating tendencies following the attention training period (Table 6). More detailed analyses showed that the difference was driven by the Awareness subscale (*p* < 0.0001, d = 0.76), with scores increasing from M = 2.40 (SD = 0.60) to M = 2.74 (SD = 0.60) and the Emotional Response subscale (*p* = 0.018, d = 0.42), reflecting increased emotional awareness and regulation while eating. No significant changes were observed in the subscales of Disinhibition, External Cues, or Distraction, indicating that these aspects of mindful eating remained relatively stable across the study period (see Table 6). Cronbach’s α for the MEQ was 0.73 (standardised α = 0.76) at baseline and 0.76 (standardised α = 0.78) post-training, indicating acceptable reliability in this sample.

### 3.5. Desire-Driven Food Behaviour and Snacking

To assess whether the attention training period could reduce desire-driven food behaviour, subjective ratings from the FCQ–T–r [52] were collected at both baseline and post-attention training. FCQ–T–r scores decreased from baseline (M = 48.2, SD = 16.1) to post-attention training (M = 45.1, SD = 14.2) (Table 7) but did not reach statistical significance (*p* = 0.069, d = 0.32). Cronbach’s α for the FCQ–T–r was 0.91 (standardised α = 0.91) at baseline and 0.90 (standardised α = 0.90) post-training, indicating excellent internal consistency.

To assess whether the attention training period affected the type of snacks consumed between meals, snack items recorded in the dietary records were categorised as healthy, unhealthy, or neutral based on definitions from the Danish National Food Institute [55,56]. A significant reduction was observed in the consumption of unhealthy snack items, with the total number per participant over the three-day period decreasing from M = 3.86 (SD = 2.6) at baseline to M = 2.74 (SD = 1.9) post-attention training, *p* = 0.005, d = 0.52 (Table 8). The proportion of participants reporting unhealthy snack consumption also declined from 73.0% to 60.4%, indicating a potential behavioural shift. In contrast, no significant changes were observed in the intake of healthy snacks (M = 2.80, SD = 2.50 vs. M = 2.43, SD = 2.00) or neutral snacks (M = 1.26, SD = 1.40 vs. M = 1.37, SD = 1.60). The proportion of participants reporting healthy snack intake remained stable (53.6% to 53.5%), while neutral snack intake increased slightly from 23.8% to 30.2% (Table 8).

### 3.6. Experience of the Attention Training Period

**Figure 3:**
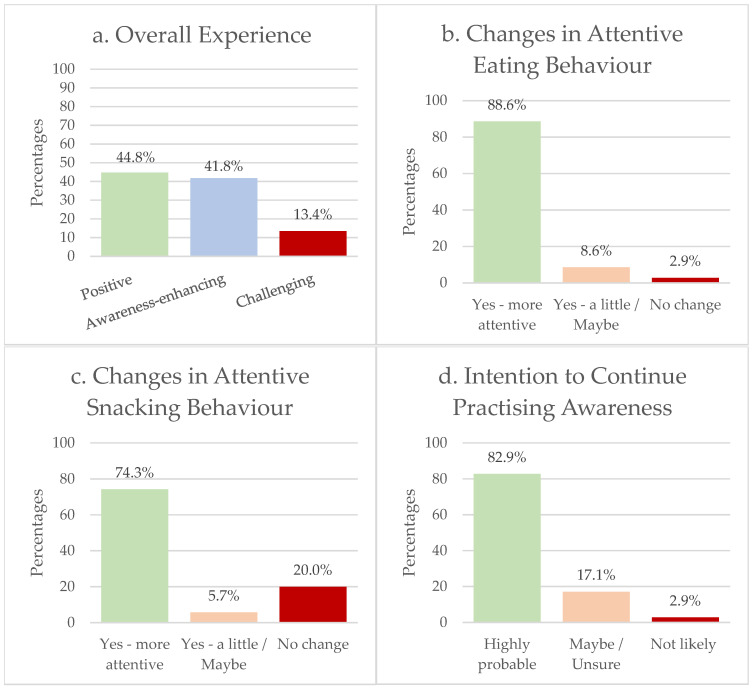
Distribution of participants’ responses regarding (**a**) reflections on the attention training period, (**b**) perceived changes in eating behaviour, (**c**) perceived changes in snacking behaviour, and (**d**) intentions to continue attention practice after completing the study (*N* = 35).

A final set of questions was administered after the attention training period to evaluate participants’ overall experience with the attention training and its perceived impact on eating behaviour. Participants’ overall experience of the attention training period was generally positive. A significant difference was found in the distribution of response types, χ^2^(2) = 33.80, *p* < 0.0001, with 44.8% of responses expressing positive experiences, 41.8% referring to increased awareness, and only 13.4% mentioning challenges (Figure 3a). This suggests that participants generally viewed the attention training as meaningful and beneficial, despite some experiencing difficulties with its implementation. In addition, participants were asked whether they had experienced changes in their eating behaviour as a result of the attention training. Responses indicated a strong perceived impact χ^2^(2) = 72.34, *p* < 0.0001, where 88.6% reported becoming more attentive and/or better handling, 8.6% reported a small or uncertain change, and only 2.9% reported no change (Figure 3b). When asked more specifically about their snacking behaviour, participants again showed a strong difference in the distribution of replies. A total of 74.3% reported that they had become more attentive or better at managing their snack intake, 5.7% reported a slight or possible change, and 20.0% reported no change. The percentages were significantly different, χ^2^(2) = 41.23, *p* < 0.0001, suggesting that the attention training had a meaningful influence on participants’ awareness and handling of snacking (Figure 3c). Finally, participants were asked whether they intended to continue practising attention and awareness in their everyday eating after the study ended. A total of 82.9% indicated that it was highly probable they would continue, 17.1% were unsure or might try, and only 2.9% said it was unlikely. The percentages were significantly different, χ^2^(2) = 56.56, *p* < 0.0001, indicating strong perceived relevance and applicability of the attention training beyond the study period (Figure 3d).

## 4. Discussion

Interoceptive and exteroceptive awareness have been proposed as important processes in the regulation of eating behaviour and may be linked to beneficial health outcomes [28,29,33]. Nevertheless, empirical research on how these abilities can be actively enhanced, and whether such enhancement supports healthier or more sustainable eating behaviours, is still at an early stage. This study examined whether written instructions delivered over a 14-day period could enhance interoceptive and exteroceptive abilities in healthy adults and promote healthier food choices. Specifically, the objectives were to study how the attention training affected: (1) interoceptive and exteroceptive abilities, (2) desire-driven eating, particularly regarding snack food consumption, and (3) the experience of the attention training period. Consistent with the initial expectations, the 14-day period of flexible yet consistent attention training appeared to enhance participants’ interoceptive and exteroceptive abilities. Participants reported significant increases in attention to various sensory experiences, as well as intuitive and mindful eating. A reduction in the consumption of unhealthy snack components was also observed. In post-study evaluations, most participants described the attention training as positive and awareness-enhancing. A large majority reported becoming more attentive in terms of their eating and snacking behaviour, and over 80% intended to continue practising awareness after the study. While not all outcomes reached statistical significance, the overall pattern of results supports the potential of interoceptive-exteroceptive attention strategies as a promising and sustainable approach to promoting healthier and more self-regulated dietary behaviours.

### 4.1. Interoceptive and Exteroceptive Eating

The present study explored whether a brief period of daily attention training could influence various aspects of eating-related awareness, spanning from internal bodily cues to external sensory inputs. Rather than finding consistent effects across all measures, the results revealed a more differentiated pattern: while several questionnaire-based outcomes showed significant improvements (e.g., intuitive eating, mindful eating and parts of EAM), other task-based measures, such as the HHT and the meal-based attention and appetite assessments, showed only marginal or no effects.

First, participants reported significant improvements in intuitive and mindful eating. Although the attention training focused on a relatively narrow form of interoceptive and sensory attention, these effects were most clearly expressed in broader constructs such as intuitive eating and mindful eating. This aligns with the conceptual scope of these measures, which assess broad patterns of eating awareness, attentional engagement and self-regulated eating behaviour, rather than narrow indices of interoceptive attention. Practising focused attention on bodily and sensory cues, even within specific tasks, may therefore translate into wider changes in how eating episodes are approached and experienced. Previous research has demonstrated that both intuitive and mindful eating can be strengthened through awareness-based strategies, even over relatively short periods [57,58,59,60], and the present findings indicate that even a brief attention training period may initiate similar shifts in everyday eating behaviour.

The attention training produced clear improvements across several exteroceptive modalities. Participants reported higher attention to taste, texture, temperature, chewing sound and combined sensory features, indicating that the attention training enhanced engagement with sensory properties that are directly experienced during oral processing. These modalities offer vivid, continuous perceptual input while eating, which likely makes them more responsive to short periods of attentional practice. The moderate effect sizes for these domains support this interpretation. By contrast, attention to appearance improved only marginally, and attention to smell and undistracted eating did not change significantly. This pattern suggests that exteroceptive attention is not uniformly malleable and that sensory modalities differ in their trainability. Smell attention showed virtually no change, consistent with evidence that olfactory perception is comparatively resistant to brief mindfulness-based interventions [61]. Mahmut and colleagues (2021) [61] reported no significant improvements in objective olfactory accuracy after short-term focused mindfulness practice, and Poellinger and colleagues (2001) [62] demonstrated that neural responses to odours habituate rapidly during prolonged stimulation. If olfactory processing is characterized by fast habituation and strong top–down influence, it is unsurprising that a short training period did not alter self-reported smell attention. The marginal improvement in appearance attention is also consistent with the idea that visual processing during eating is highly automatized. Visual evaluation of food typically occurs quickly and is strongly shaped by ingrained expectations rather than sustained perceptual monitoring. In everyday eating contexts, visual information is often processed only at the start of the meal, which may limit the degree to which appearance-related attention can be modified through brief training.

Undistracted eating also showed no significant change. This measure reflects a behavioural pattern that depends heavily on environmental and contextual factors rather than on sensory discrimination per se. Since attentional training cannot directly alter the participants’ eating environment, it is reasonable that this outcome would be less responsive than the more proximal oral sensory modalities. The broader pattern aligns with mechanistic evidence from [63]. Although their study examined neural habituation to visual and olfactory food cues rather than behavioural sensory attention during meals, their findings demonstrate that mindfulness training can modulate how food-related sensory inputs are processed over time. They observed reduced neural habituation in the mindfulness group compared to the control group, suggesting a more sustained responsiveness to sensory stimuli. However, the effects were modest, which is consistent with the idea that visual and olfactory processing are influenced by factors that may require more extended or targeted training to change [63]. This corresponds with the present findings, where the strongest changes occurred in taste, texture and other oral-sensory modalities, while appearance and smell showed limited change. Overall, these results indicate that exteroceptive attention is multidimensional and that sensory modalities vary in plasticity. Channels that offer immediate and salient perceptual feedback during eating appear to benefit most from brief attentional training, whereas modalities shaped by habitual or contextual factors show more limited change.

The lack of consistent effects across all measures may reflect differences in what each method captures (e.g., questionnaires versus behaviour). This divergence between task-based and questionnaire-based outcomes could also reflect differences in the underlying constructs being assessed: while self-report instruments may tap into more trait-like, generalised aspects of eating-related awareness, tasks such as the HHT or the meal-based ratings capture more state-dependent, momentary fluctuations. These latter outcomes may be more vulnerable to situational noise [64,65] or insufficient exposure duration, which prevents the integration of attentional shifts into moment-to-moment experiences. Although the HHT did not show significant improvement, there was a slight numerical increase in performance. This marginal improvement may indicate an emerging trend toward improved interoceptive sensitivity, although it is too small or variable to reach statistical significance in the current sample. In this sense, it aligns directionally with the questionnaire-based findings and suggests that even brief attention training may begin to influence interoceptive processing, though more sustained practice or larger samples may be needed for robust effects to manifest, as seen in the intervention carried out by [29]. The absence of significant change in the HHT, in particular, should be interpreted cautiously. Interoceptive sensitivity, as assessed by heartbeat perception tasks, has been criticised for its limited validity and vulnerability to non-interoceptive influences such as prior knowledge or cognitive estimations [66]. Thus, the marginal improvement observed cannot be taken as evidence of meaningful interoceptive change, but it also does not rule out the possibility, particularly in light of the behavioural shift in snack consumption and the improvements in self-reported intuitive and mindful eating. In this study, the HHT results are therefore best understood as preliminary signals rather than indicators of established or clinically relevant change. Future studies with longer attention training periods, larger samples and repeated assessments are needed to determine whether such early shifts can accumulate into more robust outcomes.

Similarly, participants’ attention responses during the test meal showed small and non-significant changes. This contrasts with findings by Palazzo and colleagues (2022) [29], who used a comparable task and reported clearer effects, although their study focused exclusively on exteroception in that task. The marginal changes observed in the present study may suggest that participants had begun to apply attentional strategies during eating, but that the duration of the training was not sufficient for these shifts to consolidate into more stable changes. This distinction aligns with the framework by Chun et al., (2011) [41], who emphasise that while attention can be effectively directed through instructions or training, it does not always lead to conscious awareness capable of altering behaviour. In this context, the observed changes in questionnaire-based outcomes (e.g., intuitive eating, mindful eating and parts of EAM) may reflect an initial stage of attentional shift. In contrast, behavioural tasks, like the HHT and the attention assessment, might require a more sustained transition into conscious awareness to register measurable effects. Theoretical insights from Chun et al. (2011) [41] may help interpret these findings. They distinguish between attention (the selective direction of cognitive resources) and awareness, which involves deeper conscious processing. According to their framework, sustained and repeated attentional engagement is typically required for attentional focus to evolve into awareness capable of altering behaviour. In this study, even a brief attentional training period appears to have initiated such a shift, as indicated by reductions in unhealthy snacking. This suggests that awareness may not be an all-or-nothing state but rather exists on a continuum, where partial or emerging awareness can still support meaningful behavioural outcomes.

Taken together, the results indicate that even brief and accessible attention training can begin to shape individuals’ eating-related awareness, particularly at the level of general tendencies and self-perceived behaviour. While more situational, performance-based measures may require longer or more intensive attention training to capture meaningful changes, these findings highlight the potential of everyday-compatible attentional practices. The marginal improvements in HHT performance and meal-based assessments, although not statistically robust, are noteworthy because they point in the same direction as the stronger questionnaire-based changes. This convergence suggests that the attention training period may have initiated subtle attentional shifts at both trait-like and state-dependent levels. Future studies with longer interventions, larger samples, including control groups, and repeated state-level assessments will be crucial to determine whether such small shifts can consolidate into stable behavioural and physiological changes. Future studies should further explore how duration, repetition, and contextual embedding influence the transition from attentional focus to embodied awareness and, ultimately, to sustainable changes in eating behaviour in the long term. These present findings challenge the assumption that only long-term or intensive interventions are capable of modifying eating behaviour. Instead, the present findings suggest that even brief interoceptive-exteroceptive attention strategies might contribute to changes in eating behaviour and engagement with bodily and sensory cues in real-world contexts. Future research should examine whether extending the attention training period further enhances this transition from attentional focus to sustained behavioural change and whether such shifts are maintained over time.

### 4.2. Desire-Driven Eating Behaviour and Snack Consumption

Recognising that eating is not solely governed by hunger and satiety cues [67], the present study also targeted desire- or craving-driven eating. Importantly, a significant reduction in the number of unhealthy snack components was observed following the attention training period. This shift suggests that participants may have become more aware of their snack choices, potentially favouring options more aligned with internal cues such as hunger, fullness, or physiological need rather than acting on habit, emotions, cravings, or convenience. This interpretation is consistent with previous findings linking intuitive eating to healthier dietary patterns, reduced emotional eating, and less overeating [9,68,69]. Further support for this interpretation comes from the growing literature on sensory awareness during eating. For example, prolonged oro-sensory exposure (i.e., the experience of food in the mouth) has been shown to decrease later snack intake [70]. A recent review by Lasschuijt and colleagues (2021) underscores that enhancing sensory attention may support appetite regulation and contribute to healthy weight maintenance [71].

In addition to changes in snack composition, a marginal reduction in food cravings was observed, as measured by the FCQ-T-r, from baseline to post-attention training (*p* = 0.069, d = 0.32). Although this change did not reach statistical significance, the small effect size may indicate a trend toward reduced cravings. Recent evidence indicates that cognitive as well as mindfulness oriented programmes may enhance this form of regulation, showing benefits for craving intensity, eating patterns, and in some cases also weight outcomes [72,73]. Viewed in this context, the marginal craving reduction in the present study fits the broader pattern of findings: small attentional shifts may be sufficient to initiate early changes in craving-related processes, even if they do not yet reach statistical significance. Taken together with the reduction in unhealthy snack components, this suggests that attention training may influence craving-related aspects of eating behaviour.

Future studies with longer or more intensive training periods may be better suited to detect more robust effects, as meaningful changes in dietary habits often require sustained effort over time [74]. Given that food cravings commonly influence eating behaviour [8,75] and have been linked to increased food intake and subsequent weight gain [8], reducing the impact through enhanced attention to bodily and sensory cues holds promise. This is particularly relevant when cravings are directed toward energy-dense, nutrient-poor snack products, which are typically high in fat, salt, and/or sugar yet low in essential nutrients such as dietary fibre, vitamins, and minerals [73].

A potential conceptual concern is that enhancing exteroceptive attention could be mistaken for increasing sensitivity to external food cues, which, in the case of external eating, has been associated with overeating [75]. In the present study, however, exteroception was conceptualised differently: as deliberate attention to the sensory qualities of food during eating (e.g., taste, texture, temperature), rather than automatic reactivity to environmental cues such as advertising or availability. This distinction is important, as studies indicate that paying attention to food intake and enhancing memory of meals can increase satiety and reduce subsequent intake [20,21,32]. Thus, rather than amplifying cue reactivity, exteroceptive attention training may help anchor attention to the eating episode itself and support more regulated eating. Given the very early exploratory stage of this research, it remains crucial to investigate whether some individuals may benefit more from interoceptive attention training, while others may benefit more from exteroceptive approaches. Taken together, these findings contribute to the growing body of evidence suggesting that attention-based practices, particularly those focused on bodily signals and sensory experiences, may support healthier eating behaviours in ways that are easier to integrate into everyday life.

### 4.3. Interoceptive and Exteroceptive Attention Training: Potential for Real-World Implementation

Overall, the attention training, delivered through written materials guiding participants’ focus, demonstrated promising potential as a tool for enhancing attention related to eating. When asked about their overall experience, participants generally described the attention training as positive and awareness-enhancing. Furthermore, the majority expressed a strong likelihood of continuing to practise the techniques they had learned, suggesting that such strategies may be both acceptable and sustainable in the longer term. Importantly, these findings underscore the real-world applicability of flexible, scalable approaches designed to enhance bodily and sensory attention. One likely reason for this positive reception is the programme’s simplicity. The attention training was designed as a set of flexible, easy-to-use materials, requiring little time investment and intended to be integrated into everyday routines without restricting when, how, or what individuals eat. This low-burden, self-administered format was intended to offer an alternative to more complex, multi-component approaches and may represent a pathway to supporting conscious and healthier food choices. Its simplicity and flexibility suggest potential for broader uptake and longer-term behavioural impact. At the same time, long-term feasibility remains an open question, as adherence may decline without ongoing support or reinforcement [6]. There is also potential to integrate this approach into larger multi-component interventions, where attentional strategies could complement other behavioural or lifestyle components. Such integration may help clarify whether the unique contribution of interoceptive–exteroceptive attention training lies in its standalone simplicity, or in the added value it might provide as part of more comprehensive programmes. Taken together, the present findings point to the relevance of further exploring this attentional approach, particularly with regard to its effectiveness and scalability in real-world settings.

### 4.4. Limitations

Several methodological considerations should be noted when interpreting the findings. Most importantly, the study employed a single-group pre–post design without randomisation, control groups or blinded procedures. While participants served as their own control, this design limits the ability to draw strong causal conclusions about the effectiveness of the attention training. The absence of a blank control group (i.e., participants completing only pre- and post-tests without exposure to the materials) means that environmental or repeated-testing effects cannot be excluded. Similarly, the lack of an experimental control group receiving, e.g., non-eating-related material, prevents us from ruling out expectancy or experimenter effects. Furthermore, no blinding procedures were implemented, and participants were aware that the study concerned eating behaviour, which may have influenced their responses. Taken together, these design limitations raise the possibility that some of the observed changes could be attributable to placebo or demand characteristics rather than the attention training itself.

While the 14-day attention training period may be considered relatively short, it was due to prior research interventions [43], which hypothesised that it would be sufficient to induce measurable changes in attention-related eating behaviour. Nevertheless, it cannot be ruled out that the duration of the attention training and the frequency at which the written material was used affected the results. In this study, the duration was chosen to balance ecological validity, participant compliance, and the goal of integrating attentional strategies into everyday meals without imposing extensive demands. Future studies could, however, explore the effects of longer attention training periods, as evidence suggests that the formation of new habits can take, on average, over two months of repeated behaviour in a consistent context to reach automaticity [36].

A further limitation relates to the delivery of the attention training materials outside controlled laboratory settings. Participants were instructed to read the poster daily and use the flip-cards every second day during a main meal. As a procedural check, participants also indicated how often they had used each material. Reported adherence was generally high (Table S3), with the majority using flip-cards every second day as instructed, and most participants reading the poster at least every second day. However, because these data were self-reported and the study took place in everyday environments, it cannot be certain about the accuracy of reported frequencies, the duration of use, or whether the materials were applied as intended in relation to meals. Such variability in engagement may have introduced heterogeneity to the extent to which the attentional strategies were practised. Future studies could address this by implementing more standardised delivery modes or by monitoring adherence more closely.

Another limitation concerns statistical power. Although an a priori power analysis was conducted, the present pre–post (within-subjects) design was not powered to detect small effects with high certainty. For a two-time-point within-subjects comparison, achieving power of 0.90 to detect a medium effect (Cohen’s dz ≈ 0.50) would typically require on the order of 40–45 participants, with larger samples needed for smaller effects. Thus, some null findings should be interpreted with caution. Future studies should aim for larger samples and, where appropriate, include additional measurement occasions or control groups to increase precision and internal validity.

Finally, the sample characteristics may limit the generalisability of the findings. Participants were Danish citizens and generally healthy, with normal BMI and regular engagement in physical activity, suggesting a relatively health-conscious and culturally homogeneous sample. This restricts the applicability of the results to broader populations, including individuals at higher risk for diet-related health problems or with less consistent health behaviours. To improve external validity, future studies should recruit more heterogeneous samples and assess whether attentional training strategies are equally effective across diverse health profiles and cultural contexts. Taken together, these limitations call for caution in interpreting the findings. At the same time, the study provides valuable proof-of-concept evidence that interoceptive-exteroceptive attention strategies can be implemented in everyday settings, offering a foundation for more rigorous future trials.

## 5. Conclusions

In conclusion, the findings from this study provide initial support for the use of attention strategies to enhance interoceptive and exteroceptive abilities in healthy adults and may contribute to healthier food choices. While not all outcomes reached statistical significance, the overall pattern of results suggests that interoceptive–exteroceptive strategies may initiate meaningful behavioural change. Even within a relatively short 14-day daily attention training program on interoception and exteroception using written instructions, participants reported significant increases in attention to various sensory experiences, as well as intuitive and mindful eating practices. A reduction in the consumption of unhealthy snack components was also observed. In post-study evaluations, most participants described the attention training as positive and awareness-enhancing. A large majority reported becoming more attentive in terms of their eating and snacking behaviour, and over 80% intended to continue practising awareness after the study. The outcomes emerged in the context of a daily attention training programme conducted in participants’ everyday environments, designed to require relatively little time and to avoid restrictive dietary guidelines.

Taken together, the results point to the possibility that everyday strategies focused on body and sensory attention may contribute to healthier and more sustainable eating habits. However, as this study employed a quasi-experimental, early-stage feasibility design with a single-group pre–post structure, the results should be interpreted as preliminary signals of change rather than definitive evidence of effectiveness. Future research should examine the long-term effects of attention training and investigate how such approaches can be adapted and implemented at a broader public health level.

## Figures and Tables

**Table 1:** Participant Characteristics (*N* = 35).

Characteristic	Value
**Gender**	
Female	25 (71.4%)
Male	10 (28.6%)
**BMI ^1^** (kg/m^2^)	24.82 ± 4.62
**Age** (years)	30.00 ± 11.45
**Weekly physical activity**	
Not physically active	1 (2.9%)
1–2 times a week	9 (25.7%)
3–4 times a week	12 (34.3%)
5–6 times a week	10 (28.6%)
Every day	3 (8.6%)
**Highest completed education**	
Primary school	0 (0.0%)
Secondary/high school	11 (31.4%)
Vocational education	0 (0.0%)
Short higher education (≤2 years)	1 (2.9%)
Medium higher education (3–4 years)	16 (45.7%)
Long higher education (5+ years)	7 (20.0%)
Don’t know/other	0 (0.0%)
**Primary employment status**	
Full-time employed	5 (14.3%)
Part-time employed	5 (14.3%)
Self-employed	1 (2.9%)
Student	21 (60.0%)
Unemployed	3 (8.6%)
Intern/apprentice	0 (0.0%)
On leave/sick leave/stay-at-home	0 (0.0%)
Early retiree/retiree	0 (0.0%)

Values are presented as n (%) unless otherwise indicated. ^1^ Body Mass Index = weight (kg)/height^2^ (m^2^). BMI and age are presented as mean ± standard deviation.

**Table 2:** Changes in Expression of Interoceptive and Exteroceptive Attention Related to the Consumption of a Fixed Snack Meal from Baseline to Post-Attention Training.

	Baseline	Post-Training	Mean Difference	95% CI	*p*
Exteroceptive attention	104 (81.3%)	123 (73.7%)	−0.543	[−1.343, 0.258]	0.177
Interoceptive attention	24 (18.8%)	44 (26.3%)	−0.571	[−1.156, 0.013]	0.055 ^†^

This table presents the number and proportion of meaning units reflecting participants’ attentional focus on either exteroceptive or interoceptive cues during the consumption of a standardised snack meal, as reported in the open-ended expression task. Values are shown for both baseline and post-attention training. Negative values in the mean difference column indicate higher scores for post-attention training. Effect sizes (Cohen’s d) are not reported for this analysis, as the data reflects proportions of coded meaning units rather than continuous measures. ^†^ = marginally non-significant (*p* < 0.10).

**Table 3:** Changes in Interoceptive Sensitivity Scores from Baseline to Post-Attention Training.

	Baseline	Post-Training	Mean Difference	95% CI	*p*	Cohen’s d
Interoceptive Sensitivity	0.667 ± 0.20	0.719 ± 0.20	−0.052	[−0.109, 0.005]	0.071 ^†^	0.32

Values are presented as mean ± standard deviation. Negative values in the mean difference column indicate that post-attention training yielded higher scores. ^†^ = marginally non-significant (*p* < 0.10). d = Cohen’s d (paired).

**Table 4:** Changes in the Intuitive Eating Scale (IES-2) Scores from Baseline to Post-Attention Training.

	Baseline	Post-Training	Mean Difference	95% CI	*p*	Cohen’s d
**Overall score**	3.20 ± 0.63	3.49 ± 0.51	−0.29	[−0.443, −0.128]	0.001	0.62
Eating for Physical Rather Than Emotional Reasons	3.14 ± 0.77	3.51 ± 0.66	−0.37	[−0.559, −0.184]	0.000	0.68
Reliance on Hunger and Satiety Cues	3.11 ± 0.87	3.57 ± 0.92	−0.46	[−0.698, −0.216]	0.000	0.65
Unconditional Permission to Eat	3.57 ± 0.70	3.77 ± 0.73	−0.20	[−0.417, 0.017]	0.070 ^†^	0.32

Values are presented as mean ± standard deviation. All items are measured on the Intuitive Eating Scale (IES), where higher scores indicate greater tendencies toward intuitive eating. Eating for Physical Rather Than Emotional Reasons, Reliance on Hunger and Satiety Cues, and Unconditional Permission to Eat are subscales of the IES. Negative values in the mean difference column indicate higher scores for post-attention training. ^†^ = marginally non-significant (*p* < 0.10).

**Table 5:** Changes in Exteroceptive Attention from Baseline to Post-Attention Training.

	Baseline	Post-Training	Mean Difference	95% CI	*p*	Cohen’s *d*
**Exteroceptive Attention Measures (EAM)**						
Taste Attention	61.2 ± 26.3	72.7 ± 16.5	−11.43	[−18.224, −4.633]	0.002	0.65
Texture Attention	66.6 ± 28.9	74.9 ± 22.5	−8.23	[−16.053, −0.404]	0.040	0.43
Smell Attention	64.3 ± 26.8	64.2 ± 23.5	0.03	[−7.942, 8.000]	0.994	0.00
Appearance Attention	58.0 ± 26.0	65.5 ± 24.9	−7.49	[−15.074, 0.102]	0.053 ^†^	0.34
Temperature Attention	59.9 ± 30.7	67.8 ± 20.7	−7.91	[−15.525, −0.304]	0.042	0.44
Chewing Sound Attention	34.3 ± 31.6	49.0 ± 27.1	−14.71	[−24.660, −4.769]	0.005	0.61
Combined Sensory Attention	55.9 ± 32.4	67.2 ± 23.0	−11.34	[−20.982, −1.704]	0.022	0.53
Undistracted Sensory Attention	51.9 ± 25.1	59.7 ± 22.9	−7.80	[−19.196, 3.596]	00.173	0.26
**Exteroceptive Trust Measures (ETM)**	3.08 ± 0.9	3.34 ± 1.0	−0.27	[−0.549, 0.015]	0.063 ^†^	0.29

Values are presented as mean ± standard deviation. Items are based on self-developed exteroceptive measures: EAM, ETM. Higher scores reflect greater attention to the respective sensory property and/or trust. Negative values in the mean difference column indicate that post-attention training yielded higher scores. ^†^ = marginally non-significant (*p* < 0.10).

**Table 6:** Changes in Mindful Eating Questionnaire (MEQ) Scores from Baseline to Post-Attention Training.

	Baseline	Post-Training	Mean Difference	95% CI	*p*	Cohen’s *d*
Overall score	2.49 ± 0.30	2.63 ± 0.30	−0.15	[−0.250, −0.041]	0.008	0.49
Disinhibition	2.18 ± 0.60	2.34 ± 0.70	−0.16	[−0.351, 0.036]	0.108	0.28
Awareness	2.40 ± 0.60	2.74 ± 0.60	−0.34	[−0.499, −0.187]	<0.0001	0.76
External cues	2.52 ± 0.50	2.57 ± 0.50	−0.05	[−0.204, 0.100]	0.488	0.14
Emotional response	2.84 ± 0.60	3.05 ± 0.50	−0.21	[−0.389, −0.040]	0.018	0.42
Distraction	2.50 ± 0.60	2.47 ± 0.60	0.04	[−0.202, 0.278]	0.749	0.07

Values are presented as mean ± standard deviation. All items are measured using the Mindful Eating Questionnaire (MEQ), where higher scores reflect greater mindful eating tendencies. Disinhibition, Awareness, External cues, Emotional response, and Distraction are MEQ subscales. Negative values in the mean difference column indicate higher scores for post-attention training.

**Table 7:** Changes in Trait-Level Food Cravings from Baseline to Post-Attention Training.

	Baseline	Post-Training	Mean Difference	95% CI	*p*	Cohen’s *d*
FCQ–T–r (Trait Food Cravings)	48.2 ± 16.1	45.1 ± 14.2	3.11	[−0.26, 6.49]	0.069 ^†^	0.32

Values are presented as mean ± standard deviation. Higher mean scores indicate stronger trait-level food cravings. Positive values in the mean difference column indicate lower scores for post-attention training. ^†^ = marginally non-significant (*p* < 0.10).

**Table 8:** Changes in Snack Intake by Perceived Healthiness from Baseline to Post-Attention Training.

	Baseline	Post-Training	Mean Difference	95% CI	*p*	Cohen’s *d*
Unhealthy snacks	3.86 ± 2.6	2.74 ± 1.9	1.11	[0.37, 1.86]	0.005	0.52
Healthy snacks	2.80 ± 2.5	2.43 ± 2.0	0.37	[−0.42, 1.16]	0.347	0.18
Neutral snacks	1.26 ± 1.4	1.37 ± 1.6	−0.11	[−0.52, 0.29]	0.571	0.10

Values are presented as mean ± standard deviation. Snack meal data were derived from three-day dietary records and categorised into healthy, unhealthy, and neutral items based on definitions from the Danish National Food Institute. Items within the same snack occasion could fall into multiple categories. *p*-values are based on paired *t*-tests.

## Data Availability

The original contributions presented in the study are included in the article/Supplementary Materials, further inquiries can be directed to the corresponding author.

## Supplementary Materials

The following supporting information can be downloaded at: https://www.mdpi.com/article/10.3390/foods14234078/s1. Figure S1 Attention training material: Informational flyer. The figure shows the flyer used as part of the attention training in the study. The left panels display the original Danish version provided to participants, while the right panels show the corresponding English. Figure S2 Attention training material: Poster. The figure shows the poster used as part of the attention training in the study. The left panels display the original Danish version provided to participants, while the right panels show the corresponding English. Figure S3 Attention Training Material: Flip Cards: Image of the original flip cards used as part of the attention training in the study. Table S1 Attention Training Material: Flip Cards: The table shows the phases in the original Danish version alongside their corresponding English translations. Table S2 Overview of Questionnaire Phrasing (Danish/English) and Response Variables Used in the Study. Table S3 Adherence and perceived supportiveness of the attention training materials across the 14-day attention training. Table S4 Mean ± SD delta values (Δ1 and Δ2) for appetite and sensory-specific desire ratings.
