# Supplementary material for: Effects of Interoceptive and Exteroceptive Attention Training on Desire-Driven Eating in Healthy Adults: A Quasi-Experimental Early-Stage Feasibility Study
Source: Foods. 2025 Nov 27;14(23):4078. doi: 10.3390/foods14234078 (PMC12692140; doi:10.3390/foods14234078)
Supplement: Supplementary file 1 [file foods-14-04078-s001.zip › foods-3971344-supplementary.pdf]

Supplementary Material

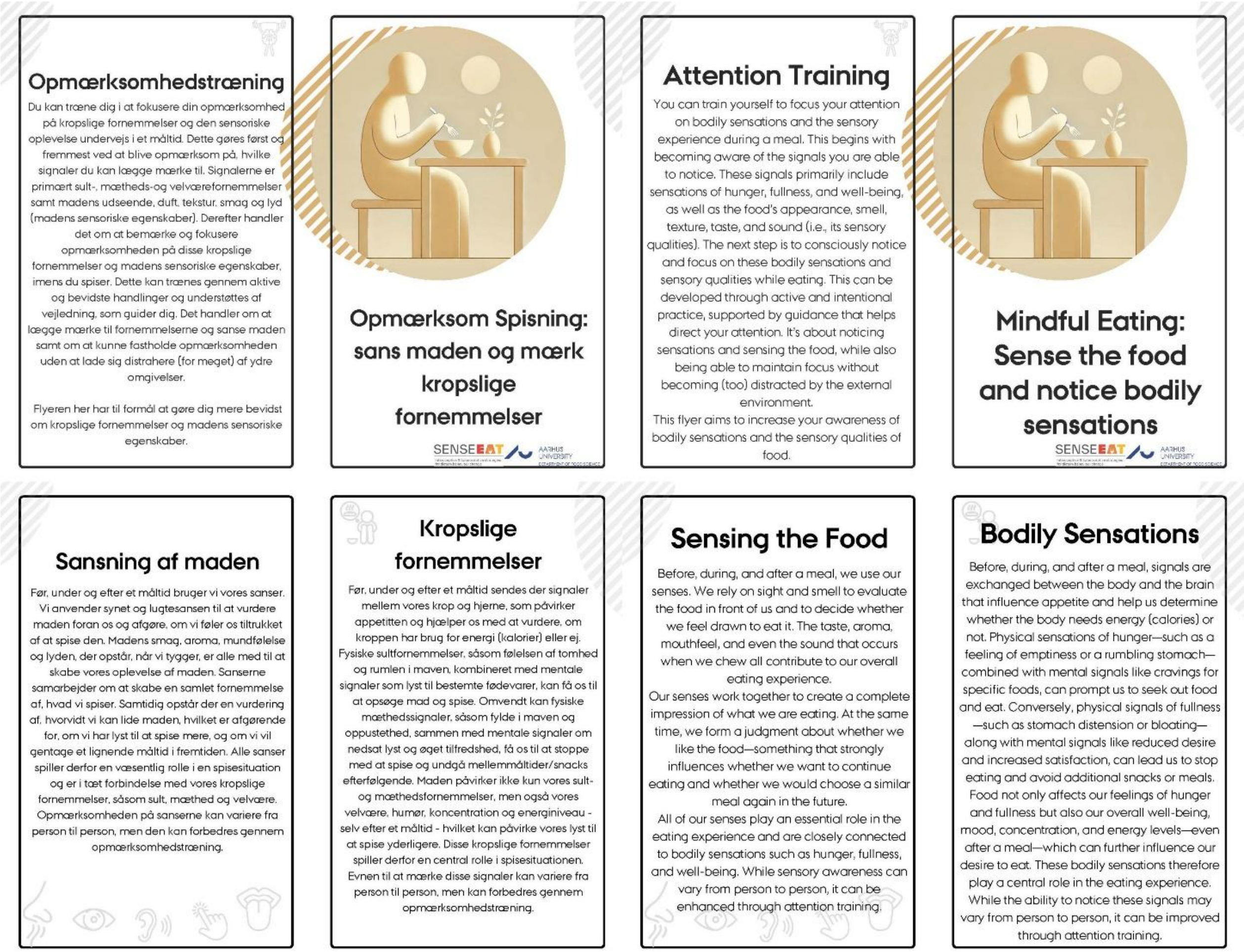

Figure S1. Attention training material: Informational flyer. The figure shows the flyer used as part of the attention training in the study. The left panels display the original Danish version provided to participants, while the right panels show the corresponding English

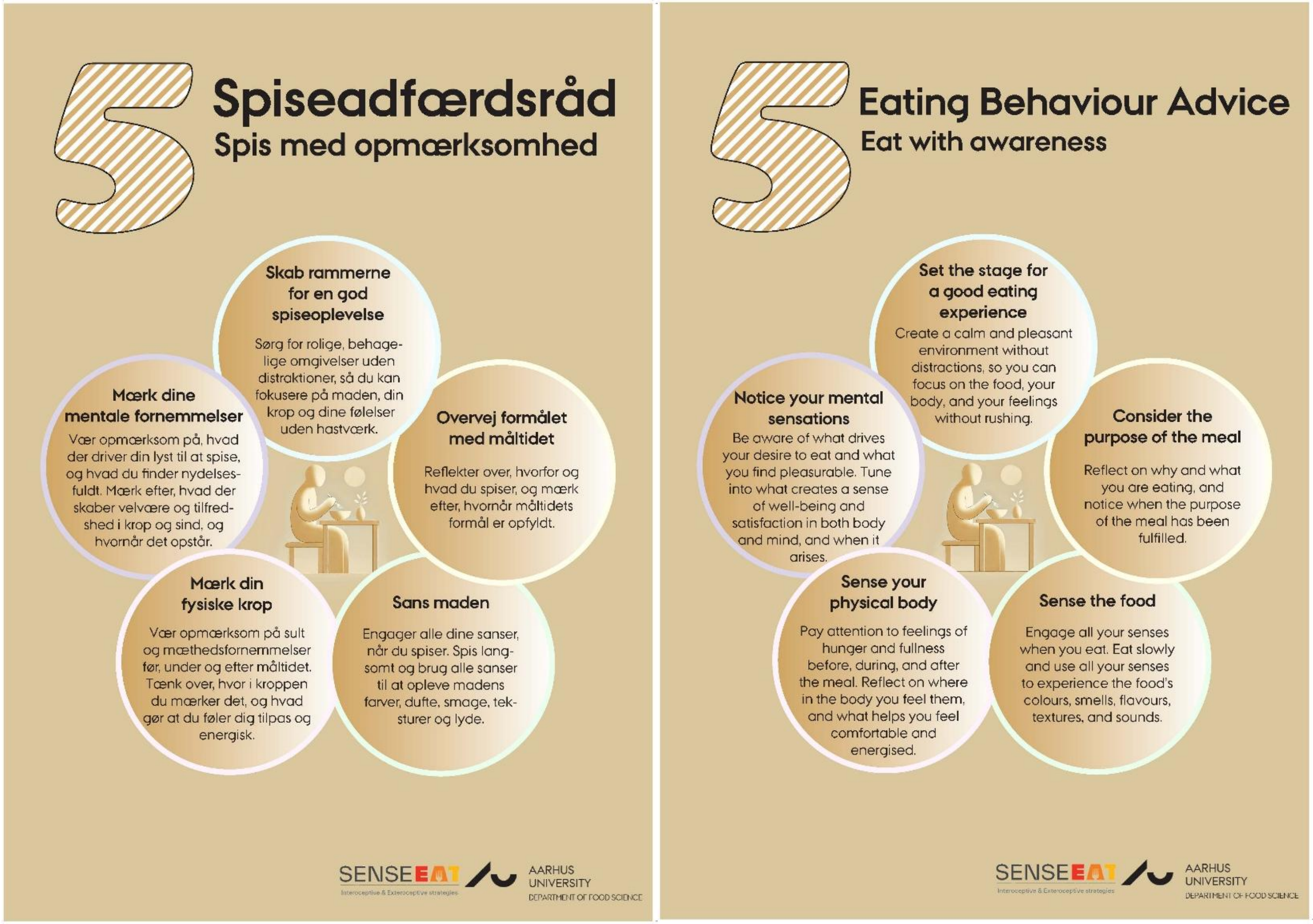

Figure S2. Attention training material: Poster. The figure shows the poster used as part of the attention training in the study. The left panels display the original Danish version provided to participants, while the right panels show the corresponding English

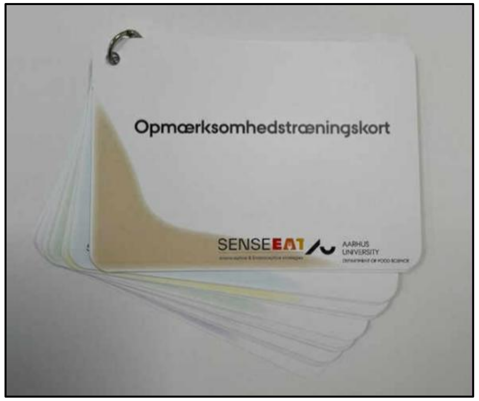

Figure S3. Attention Training Material: Flip Cards: Image of the original flip cards used as part of the attention training in the study.

Table S1. Attention Training Material: Flip Cards: The table shows the phases in the original Danish version alongside their corresponding English translations.

**Flip Cards Phrases**		
	**Danish (original)**	**English**
Page 1 (frontpage)	**Opmærksomhedstræningskort**	**Attention Training Card**
Page 2	**Skab rammerne for en god spiseoplevelse**	**Set the stage for a good eating experience**
Page 3	Før og mens du spiser, skab en ramme for måltidet, som understøtter, at du kan være opmærksom på maden samt dine kropslige og mentale fornemmelser. Når du spiser, så fjern unødvendige distraktioner såsom tv, tablets og smartphones, og sørg for at have tilstrækkelig tid til at spise.	Before and during your meal, create an environment that supports your ability to pay attention to the food, as well as your bodily and mental sensations. When eating, remove unnecessary distractions such as the TV, tablets, and smartphones, and make sure you have enough time to eat without feeling rushed.

Page 4	Før og mens du spiser, sid behageligt og tag et par dybe indåndinger for at slappe af i din krop og dine tanker. Ret fokus på maden og fornemmelserne i din krop, og genopret fokus undervejs i måltidet, hvis distraktioner skulle forekomme.	Before and during your meal, sit comfortably and take a few deep breaths to relax your body and mind. Direct your attention to the food and the sensations in your body, and gently bring your focus back if distractions arise during the meal.
Page 5	**Overvej formålet med måltidet**	**Consider the purpose of the meal**
Page 6	Før du spiser, så spørg dig selv, hvilket formål måltidet skal tjene. Er det en følelse af sult, fordi kroppen mangler energi, der motiverer dig, eller er det andre ting såsom en fristelse, humør eller vane, der får dig til at spise?	Before you eat, ask yourself what purpose the meal is meant to serve. Is it a feeling of hunger—a signal that your body needs energy—that motivates you, or is it something else, such as temptation, mood, or habit, that drives you to eat?
Page 7	Imens du spiser, så mærk efter, hvornår formålet med måltidet er opfyldt. Har kroppen fx fået den energi, den man-glede? Er din lyst blevet tilfredsstillet, eller er dit humør blevet løftet? Husk at tage små pauser undervejs for at fornemme, hvordan du har det, og hvordan maden påvirker dig, og afslut måltidet, når formålet er nået.	While eating, notice when the purpose of the meal has been fulfilled. Has your body, for example, received the energy it needed? Has your craving been satisfied, or has your mood improved? Remember to take small pauses throughout the meal to sense how you feel and how the food is affecting you, and finish eating when the purpose has been met.
Page 8	**Sans maden**	**Sense the food**
Page 9	Fokuser din opmærksomhed på maden. Fornem, hvordan den ser ud, dufter, føles i munden, smager, og hvordan den lyder, når du tygger. Mens du spiser, prøv at spise og tygge din mad langsommere, end du plejer.	Focus your attention on the food. Notice how it looks, smells, feels in your mouth, tastes, and sounds as you chew. While eating, try to eat and chew more slowly than you normally do.
Page 10	Fokuser nu mere detaljeret på smagen. Er der ingredienser, der bidrager med noget sødt, salt og fedt i måltidet? Fornem, hvor intenst det smager.	Now focus more specifically on the taste. Are there ingredients that contribute sweetness, saltiness, or fattiness to the meal? Notice how intense the flavours are.
Page 11	**Mærk din fysiske krop**	**Sense your physical body**
Page 12	Fokuser din opmærksomhed på din krop og de fysiske signaler, den sender. Mærk, hvordan den signalerer sult. Føles det som en rumlende mave, lav energi eller noget andet? Og mærk efter, hvor sulten føler du dig?	Focus your attention on your body and the physical signals it sends. Notice how it signals hunger—does it feel like a rumbling stomach, low energy, or something else? And tune into how hungry you actually feel.

Page 13	Imens du spiser, vær opmærksom på, om de fysiske sultfornemmelser forsvinder og overtages af mæthedsfornemmelser. Mærk, hvordan din krop signalerer mæthed. Føles det som fysisk velvære, mad i maven, energi eller ro i kroppen?	While eating, pay attention to whether the physical sensations of hunger begin to fade and are replaced by feelings of fullness. Notice how your body signals that it's becoming full—does it feel like physical comfort, food in the stomach, increased energy, or a sense of calm in the body?
Page 14	**Mærk dine mentale fornemmelser**	**Notice your mental sensations**
Page 15	Fokuser din opmærksomhed på din mentale lyst til mad. Fornem, hvordan den signalerer lyst, og tænk over, hvad der bidrager til den lyst til mad (eller mangel på samme), som du mærker.	Focus your attention on your mental desire for food. Notice how this desire shows up, and reflect on what might be contributing to the urge to eat—or the lack of it—that you're experiencing.
Page 16	Imens du spiser, læg mærke til, om din lyst bliver tilfredsstillet. Mærk, hvordan madens smag, fx salt, sødt og fedme, og mæthed tilfredsstiller din lyst, giver nydelse og evt. påvirker dit humør.	While eating, notice whether your desire is being satisfied. Pay attention to how the flavours—such as saltiness, sweetness, and fattiness—and the feeling of fullness satisfy your desire, bring pleasure, and perhaps even influence your mood.

Table S2. Overview of Questionnaire Phrasing (Danish/English) and Response Variables Used in the Study.

Appetite and Sensory-Specific-Desire Sensations[1]

Category	Question in Danish	Question in English	Danish reply scale	English reply scale
Hunger	Hvor sulten er du?	How hungry are you?	10cm VAS skala: Overhovedet ikke – Ekstremt meget	10cm VAS scale: Not at all – Extremely
Fullness	Hvor mæt er du?	How satiated are you?		
Sensory Specific Desires	Hvor meget har du lyst til at spise noget krydret?	How much do you desire to eat something spicy?		

Hvor meget har du lyst til at spise noget surt?	How much do you desire to eat something sour?
Hvor meget har du lyst til at spise noget fedt?	How much do you desire to eat something fatty?
Hvor meget har du lyst til at spise noget salt?	How much do you desire to eat something salty?
Hvor meget har du lyst til at spise noget sødt?	How much do you desire to eat something sweet?
Hvor meget har du lyst til at spise noget bittert?	How much do you desire to eat something bitter?

Exteroceptive Attention Measures (EAM)[2]

Category	Question in Danish	Question in English	Danish reply scale	English reply scale
**Intro**	I dette skema vil der være nogle spørgsmål vedrørende din opmærksomhed på maden eller fødevaren, du indtager. Læs hvert udsagn og bedøm, hvordan det forholder sig for dig, ved at lave en markering på skalaen.	In this questionnaire, there will be some questions regarding your attention to the food or the food items you consume. Read each statement and assess how it applies to you by making a mark on the scale.		
**Taste Attention**	I hvor høj grad er du opmærksom på forskellige smage i den fødevare/mad,	To what extent do you pay attention to different tastes in the food you	10cm VAS skala: Overhovedet ikke	10cm VAS scale: Not at all attentive

	du indtager, såsom sødt, salt, surt, bittert og umami?	consume, such as sweet, salty, sour, bitter, and umami?	opmærksom – Ekstremt opmærksom	– Extremely attentive
**Texture Attention**	I hvor høj grad er du opmærksom på forskellige teksturer i den fødevare/mad, du indtager, såsom sprødhed, grynethed eller cremethed?	To what extent do you pay attention to different textures in the foods you consume, such as crispness, gritty, or creaminess?		
**Smell Attention**	I hvor høj grad er du opmærksom på forskellige lugte/dufte i den fødevare/mad, du indtager?	To what extent do you pay attention to different smells in the food you consume?		
**Appearance Attention**	I hvor høj grad er du opmærksom på udseendet af den fødevare/mad, du indtager?	To what extent do you pay attention to the appearance of the food you consume?		
**Temperature Attention**	I hvor høj grad er du opmærksom på temperaturen af den fødevare/mad, du indtager?	To what extent do you pay attention to the temperature of the foods you consume?		
**Chewing Sound Attention**	I hvor høj grad er du opmærksom på, hvordan fødevaren/maden lyder, når du tygger den?	To what extent do you pay attention to how the food sounds when you chew it?		
**Combined Attention on Sensory Properties**	I hvor høj grad er du opmærksom på kombinationen af de sensoriske egenskaber ved den fødevare/mad, du	To what extent do you pay attention to the combination of different sensory properties of the food you		

	indtager, såsom samspillet mellem smag, tekstur, duft og udseende?	consume, such as the interplay of taste, texture, smell, and appearance?		
**Attention to Sensory Properties Without Being Distracted**	I hvor høj grad er du i stand til at være opmærksom på fødevarens/madens sensoriske egenskaber før og under indtagelse, uden at lade dig distrahere af eksterne faktorer eller stimuli?	To what extent are you able to pay attention to the sensory properties of the food before and during consumption without being distracted by external factors or stimuli?	10cm VAS skala: Jeg bliver overhovedet ikke distraheret – Jeg bliver ekstremt nemt distraheret	10cm VAS scale: I don't get distracted at all - I get distracted extremely easily

Exteroceptive Trust Measures (ETM)[3]

Category	Question in Danish	Question in English	Danish reply scale	English reply scale
**Intro**	I dette spørgeskema vil du blive spurgt om din oplevelse af lyst til mad i forbindelse med måltider, og om du har tillid til, at denne lyst er drevet af kroppens behov.	In this questionnaire, you will be asked about your experience of desires in connection with meals and whether you trust that these cravings are driven by your body’s needs.	5-point skala: Stærkt uenig (1), Uenig (2), Neutral (3), Enig (4), Stærkt enig (5)	5-point scale: Strongly Disagree (1), Disagree (2), Neutral (3), Agree (4), Strongly Agree (5)
**Trust**	Jeg stoler på, at min oplevede lyst til specifikke smage eller teksturer er kroppens måde at fortælle mig, hvornår jeg skal spise	I trust that my perceived desire for specific tastes or textures is my body's way of telling me when to eat.		
**Trust**	Jeg stoler på, at min oplevede lyst til specifikke smage eller teksturer er kroppens måde at fortælle mig, hvad jeg bør spise	I trust that my perceived desire for specific tastes or textures is my body's way of telling me what to eat.		

**Trust**	Jeg stoler på, at min oplevede lyst til specifikke smage eller teksturer er kroppens måde at fortælle mig, hvor meget jeg bør spise	I trust that my perceived desire for specific tastes or textures is my body's way of telling me how much to eat.
**Reliance**	Jeg regner med, at jeg oplever en øget lyst til specifikke smage eller teksturer, når kroppen har behov for, at jeg begynder at spise	I rely on that I experience an increased desire for specific tastes or textures when my body needs me to start eating.
**Reliance**	Jeg regner med, at jeg oplever en nedsat lyst til mad, når kroppen har behov for, at jeg stopper med at spise	I rely on that I experience a decreased desire for food when my body needs me to stop eating.
**Trust**	Jeg stoler på, at min nedsatte lyst til specifikke smage eller teksturer fortæller mig, hvornår jeg skal stoppe med at spise	I trust that my decreased desire for specific tastes or textures tells me when to stop eating.

Socio-Demographic Questions – asked only post-attention training

Category	Question in Danish	Question in English	Danish reply scale	English reply scale
**Intro**	De næste spørgsmål handler om dine socio-demografiske forhold.	The next questions are about your socio-demographic background.		
**Gender**	Hvad er dit biologiske køn ved fødsel?	What is your biological gender at birth?	- Kvinde<br>- Mand	- Female<br>- Male
**Age**	Hvad er din alder?	What is your age?	Angiv venligst din alder I hele tal	Please indicate your age in whole numbers
**Physically activity**	I gennemsnit, hvor ofte er du fysisk aktiv* på en uge?	On average, how often are you physically active* in a week?	- Jeg er ikke fysisk aktiv	- I am not physically active

	**Fysisk aktivitet er alle former for bevægelse, der øger energiomsætningen. Det kan være ustruktureret aktivitet og mere bevidst målrettet regelmæssig fysisk aktivitet (træning). Fysisk aktivitet kan med andre ord godt indgå som en del af hverdagen og ens vanlige gøremål.*	**Physical activity includes all forms of movement that increase energy expenditure. It can be an unstructured activity or a more consciously targeted regular physical activity (exercise). In other words, physical activity can be part of everyday life and one's usual activities*	- 1-2 gange om ugen<br>- 3-4 gange om ugen<br>- 5-6 gange om ugen<br>- Hver dag	- 1-2 times a week<br>- 3-4 times a week<br>- 5-6 times a week<br>- Every day
**Educational level**	Hvad er dit højeste fuldførte uddannelsesniveau?	What is your highest completed education?	- Folkeskole / grundskole<br>- Gymnasial uddannelse<br>- Erhvervsfaglig uddannelse<br>- Kort videregående uddannelse f.eks. erhvervsakademiuddannelse (2 år)<br>- Mellemlang videregående uddannelse (3-4 år)<br>- Lang videregående uddannelse (5+ år)<br>- Ved ikke / andet	- Primary school/elementary school<br>- Secondary/ High-school education<br>- Vocational education<br>- Short higher education (2 years)<br>- Medium higher education (3-4 years)<br>- Longer higher education (5+ years)<br>- Don't Know / Other

**Employment**	Hvad er din primære beskæftigelsesstatus?	What is your primary employment status?	- Fuldtidsansat<br>- Deltidsansat<br>- Praktikant/lærling<br>- Selvstændig<br>- Studerende<br>- Arbejdsløs<br>- Førtidspensionist/pensionist<br>- Hjemmegående<br>- Orlov/sygemeldt	- Full-time employed<br>- Part-time employed<br>- Intern/apprentice<br>- Self-employed<br>- Student<br>- Unemployed<br>- Early retiree/retiree<br>- Stay-at-home<br>- leave/sick leave

Final participant reflections and evaluation – asked only post-attention training

Category	Question in Danish	Question in English	Danish reply scale	English reply scale
**Intro**	De sidste spørgsmål handler om din oplevelse af at deltage i studiet. Besvar venligst alle spørgsmål så åbent og ærligt som muligt. Husk, at der ikke er nogen rigtige eller forkerte svar.	The final questions are about your experience of participating in the study. Please answer all questions as openly and honestly as possible. Remember, there are no right or wrong answers.		
**General Experience**	Hvordan vil du beskrive din overordnede oplevelse af opmærksomhedstræningsperioden i studiet?	How would you describe your overall experience of the attention training period in the study?	Åbent spørgsmål (fri tekst)	Open-ended (free text)

**Behavioural Changes**	Nu hvor du har trænet din opmærksomhed i to uger, har du oplevet nogle ændringer efter at have gennemført opmærksomhedsøvelserne i forhold til din adfærd før og under måltider?	Now that you have practiced your awareness for two weeks, have you noticed any changes after completing the awareness exercises in relation to your behavior before and during meals?	Åbent spørgsmål (fri tekst)	Open-ended (free text)
**Snack Consumption**	På hvilke måder, hvis nogle, har fokus på kropslige signaler og den sensoriske oplevelse under spisning påvirket, hvordan du opfatter og håndterer dit snackforbrug?	In what ways, if any, has focus on bodily signals and the sensory experience while eating influenced how you perceive and manage your snack consumption?	Åbent spørgsmål (fri tekst)	Open-ended (free text)
**Continuation of Practice**	Hvor sandsynligt er det, at du vil fortsætte med at øve dig i at være opmærksom på de sensoriske egenskaber ved den mad, du spiser, og dine kropslige fornemmelser efter denne undersøgelse? Forklar venligst hvorfor/hvorfor ikke.	How likely is it that you will continue practising paying attention to the sensory characteristics of the food you consume and your bodily sensations after this study? Please explain why/why not.	Åbent spørgsmål (fri tekst)	Open-ended (free text)

Table S3. Adherence and perceived supportiveness of the attention training materials across the 14-day attention training.

**Usage Frequency***	**Flip-cards**	**Poster**
Daily (all 14 days)	4 (11.4%)	15 (42.9%)
Every second day (≈ 7 days)	23 (65.7%)	12 (34.3%)
A few times per week (4–6 days)	8 (22.9%)	6 (17.1%)
Less than a few times per week (1–3 days)	0 (0%)	2 (5.7%)
Did not use	0 (0%)	0 (0%)
**Perceived supportiveness***	**First seven days**	**Last seven days**
Flip-cards	65 ± 26.8	55 ± 23.1
Poster	63 ± 23.6	56 ± 24.4

*Values are presented as mean ± standard deviation.

Table S4. Mean ± SD delta values (Δ1 and Δ2) for appetite and sensory-specific desire ratings.

	**Δ1 (Baseline) Mean ± SD**	**Δ2 (Post-attention training) Mean ± SD**
Hunger	-17.33 ± 12.46	-21.14 ± 27.50
Satiety	20.82 ± 15.28	24.46 ± 28.35
Desire for spicy	-8.94 ± 17.41	-6.23 ± 11.81
Desire for sour	-12.88 ± 24.01	-10.54 ± 17.53
Desire for fatty	-10.39 ± 11.76	-12.91 ± 16.59
Desire for salty	-11.36 ± 21.85	-12.66 ± 14.30
Desire for sweet	-19.15 ± 28.19	-16.57 ± 22.54
Desire for bitter	-1.45 ± 17.21	-8.11 ± 10.37
